# Amplifying the role of knowledge translation platforms in the COVID-19 pandemic response

**DOI:** 10.1186/s12961-020-00576-y

**Published:** 2020-06-03

**Authors:** Fadi El-Jardali, Lama Bou-Karroum, Racha Fadlallah

**Affiliations:** 1 Department of Health Management and Policy, Faculty of Health Sciences, American University of Beirut, Beirut, Lebanon; 2 Knowledge to Policy (K2P) Center, Faculty of Health Sciences, American University of Beirut, Beirut, Lebanon; 3 Department of Health Research Methods, Evidence, and Impact, McMaster University, Hamilton, Ontario Canada

**Keywords:** COVID-19 pandemic, knowledge translation, evidence-informed policy-making, evidence, research, data

## Abstract

The COVID-19 pandemic presents the worst public health crisis in recent history. The response to the COVID-19 pandemic has been challenged by many factors, including scientific uncertainties, scarcity of relevant research, proliferation of misinformation and fake news, poor access to actionable evidence, time constraints, and weak collaborations among relevant stakeholders. Knowledge translation (KT) platforms, composed of organisations, initiatives and networks supporting evidence-informed policy-making, can play an important role in providing relevant and timely evidence to inform pandemic responses and bridge the gap between science, policy, practice and politics. In this Commentary, we highlight the emerging roles of KT platforms in light of the COVID-19 pandemic. We also reflect on the lessons learned from the efforts of a KT platform in a middle-income country to inform decision-making and practice during the COVID-19 pandemic. The lessons learned can be integrated into strengthening the role, structures and mandates of KT platforms as hubs for trustworthy evidence that can inform policies and practice during public health crises and in promoting their integration and institutionalisation within the policy-making processes.

## Background

The world is facing the worst public health crisis in recent history. The coronavirus disease (COVID-19) pandemic has directly and indirectly affected more than four million people across the globe, disrupting health systems and economies [1]. It has placed unprecedented pressure on decision-makers at all levels to make rapid decisions, oftentimes in the face of uncertainty and with long-term consequences on the lives of millions of people [1, 2].

Using the best available research evidence and data to guide public health and health systems decisions is integral to an effective and efficient response in public health emergencies [2–4]. In crises of such devastating scale and intensity, this could make the difference between life and death [5]. Despite the breadth of available research evidence on epidemic preparedness and on different preventive measures, such as travel restriction [6], school closures [7], disease surveillance networks [8] and quarantine [9], this evidence was inconsistently used in informing decisions [10]. Even in countries where public health expertise was available, governments did not leverage this expertise to better mitigate trade-offs and guide the policy response in the face of uncertainties [11]. Instead, many governments reverted to a top-down approach to decision-making with political and economic considerations and other contextual factors taking precedence over research and health directives, further exposing populations to risks [10, 12].

Organisations, initiatives and networks that support evidence-informedpolicy-making can play an important role in providing relevant and timely evidence to inform pandemic responses and bridge the gap between science, policy and politics. An organised form of these aforementioned entities, known as knowledge translation (KT) platforms, brings decision-makers, researchers, practitioners, civil society groups and other stakeholders together to facilitate the process of translating evidence into policy and action by aligning research topics with policy priorities, responding to pressing issues through developing policy briefs, rapid responses and evidence summaries, and convening dialogues to guide policy formulation and implementation, taking into consideration local and political context [13–15].

The role of KT platforms in pandemic responses is more important than ever, particularly in low- and middle-income countries, where public health and health systems are already overburdened and under-resourced, thus posing additional challenges to effective response. In this Commentary, we highlight the emerging roles of KT platforms in light of the COVID-19 pandemic. We also reflect on the lessons learned from a KT platform effort in a middle-income country to inform decision-making and practice during the COVID-19 pandemic. Lessons learned can be integrated into strengthening the role, structures and mandates of KT platforms as hubs for trustworthy evidence that can inform policies and practice during public health crises.

## Emerging roles of KT platforms in pandemic response

KT platforms are uniquely positioned to help facilitate a rapid evidence-informed response during public health crises in different ways, as outlined below.

### Engaging decision-makers and stakeholders in setting priorities

A key starting point to inform decision-making processes is to identify policy- and practice-relevant priorities. Engaging decision-makers in priority-setting can increase the acceptability of evidence and foster its utilisation in the decision-making process [16]. KT platforms can play an important role in prioritising relevant issues, mapping knowledge gaps, and aligning research and evidence synthesis topics with policy needs during a public health crisis. Providing decision-makers and practitioners with policy- and practice-relevant evidence can help inform critical decisions and contribute to strengthening health systems and improving population health. It can also help overcome the scarcity of resources by pursuing and acting upon high priority issues that are likely to have a significant impact on knowledge, policy or practice [17, 18].

### Synthesising the best available evidence: separating the wheat from the chaff

With the rapid spread of COVID-19 around the globe, decision-makers and practitioners are also required to mount a rapid response. This necessitates timely and relevant evidence to inform prevention, control and mitigation measures. Evidence includes not only findings from research but also other forms of knowledge such as local data, surveillance data, guidelines, national and international agency reports as well as media reports [19]. However, the speed with which the information is being generated during the public health crisis is unprecedented — decision-makers and practitioners are swamped with a tsunami of information, both reliable and unreliable, making it challenging to remain abreast of the rapidly evolving evidence base and to filter the type and structure of evidence that would be of most value to inform decision-making and practice.

Acknowledging the tension between rigor and speed, KT platforms can harness this large body of knowledge through synthesis to inform policy and practice. Specifically, they can filter current best available evidence from unsubstantiated or non-scientifically supervised sources and combine the results of multiple relevant studies while taking into consideration the quality of studies and biases in the existing evidence base. In doing so, they can provide decision-makers and practitioners with relevant, reliable and accessible evidence syntheses that address priority issues in a timely and transparent manner.

### Contextualising and disseminating actionable evidence to target audience: turning the noise into music

The availability of evidence is a necessary but insufficient condition for evidence-informed response during public health crisis; evidence must reach people in policy and operational positions in an actionable form and with minimal time lag and, when used, should be applied effectively [20]. Often, relevant evidence exists but decision-makers are unaware or unable to interpret or apply it to their own context [21]. Beyond evidence from research, decision-makers are also influenced by numerous forces, including institutional constraints, political context, interest group pressures, personal convictions and values, and external factors such as economic recession.

KT platforms recognise that a ‘one-size-fits-all’ approach for responding to public health crises may not be appropriate and, therefore, they provide context-specific and actionable evidence to enable countries to adapt global solutions to local needs and realities. Specifically, they can produce a range of KT products by combining insights from multiple sources (systematic/rapid reviews, primary studies, local date and tacit knowledge) in a user-friendly format and plain language and tailoring the findings to local context. To further foster the use of evidence in policies and practice, they can leverage a range of uptake activities to disseminate the products to target audience and support the use of that evidence to its full potential. Additionally, they can maintain repositories of product pipelines that can serve as a ‘one-stop shop’ freely accessible by the different stakeholders to support evidence use in a timely manner.

### Promoting trust and countering misinformation

“*We’re not just fighting an epidemic; we’re fighting an infodemic*” – Dr. Tedros Adhanom Ghebreyesus, WHO Director General, 15 February 2020.

While infodemics (or overflow of information) are not a novel phenomenon, the speed and reach of the COVID-19 infodemic and fake news has been unprecedented [22]. This makes it challenging for people to find trustworthy sources and reliable information to guide them when they need it. It can also hamper the effectiveness of a pandemic response [5]. KT platforms are uniquely positioned to serve as a credible hub that provides trustworthy evidence that can be acted upon by policy-makers, stakeholders, citizens and media in times of crisis.

### Providing platforms for cross-sectoral dialogue: breaking down silos

The scope and intensity of public health crisis means that no single agency can work alone to effectively control and mitigate its impact. Governments need to collaborate and shape the collective response through multi-sectoral actions that involve the public, private and civil society sectors [23].

KT platforms can facilitate the coordinated and multi-disciplinary approach needed to inform policy and practice during a public health crisis. They can provide a platform (physical or web-enabled, depending on the crisis) that brings together the different stakeholders (e.g. policy-makers, researchers, practitioners, civil society organisations) from different sectors (e.g. health, education, social, economy) to shape policy problems, increase mutual understanding of challenges faced, deliberate about policy and practice solutions (including their benefits, potential harms, costs and uncertainties), and discuss implementation considerations. They can also serve as a neutral communication channel for real-time exchange of relevant information, ideas, concerns and best practices as the crisis unfolds. In doing so, they enable stakeholder group interaction, redistribution of power resources and evidence-informed deliberations alongside contextual factors, such as values, beliefs, interests or political goals, and the strategies of the different stakeholders [24]. Importantly, they can ensure that the voices of those who are most affected by a public health crisis are meaningfully included in the decision-making process.

### Monitoring and evaluating policy response

During the 2009 H1N1 influenza pandemic, evaluations of implemented response measures were hindered by the lack of planning for this activity, political sensitivities and legal issues. Additionally, little attention was paid to the recovery phase and to the transition to seasonal influenza [25]. This posed a missed opportunity to learn what worked for whom and in what circumstances in order to generate valuable lessons for informing future pandemics like COVID-19. KT platforms can help in monitoring the effectiveness of prevention and mitigation measures during a crisis and in assessing the impact on different population groups to guide a response effort that is more inclusive, equitable and responsive to the contextual needs. In doing so, they can play an important role in assessing the immediate and longer-term health, social and economic effects of the pandemic, which is critical to inform the transition to the post-pandemic phase.

## Lessons learned from a KT platform

Below, we reflect on the experience and lessons learned so far from the Knowledge to Policy (K2P) Center, a WHO Collaborating Centre for Evidence-Informed Policymaking and Practice. Established within a university setting in Lebanon (a middle-income country), K2P Center seeks to bridge the gap between science, policy and politics by making research evidence more accessible to a broader range of stakeholders, building institutional capacities for evidence-informedpolicy-making and seizing opportunities to advocate and influence policy outcomes. The Center’s response to COVID-19 builds on years of work in raising the awareness of policy-makers, stakeholders, civil society organisations and media on the importance of evidence in informing decision-making, developing their capacities in accessing and using evidence, raising demand for evidence, building trust and establishing critical linkages. With the pandemic still unfolding, new lessons will likely emerge, making this a learning platform and an evolving living experience.

### Activate rapid response services

During a public health crisis, decision-makers are under tremendous pressure to respond urgently to demonstrate capability and meet public health needs. The time limitation is a critical barrier to evidence use during a crisis, thus necessitating the provision of evidence in a timely manner to decision-makers. In this regard, the rapid response service presents a key element in the response to the COVID-19 pandemic. It can respond to urgent requests from decision-makers and stakeholders by delivering relevant and high-quality evidence in short periods of time, ranging from 3 to 30 days. Rapid response products use systematic and transparent methods to search, synthesise and appraise the existing evidence base (giving precedence to existing systematic/rapid reviews when possible) while tailoring the implications to local context in order to support policy and systems decisions. They also utilise user-friendly formats and plain language to facilitate the uptake of evidence in decision-making.

KT platforms should have the capability and flexibility to switch to rapid response mode during a crisis and tailor their suite of services to address the various aspects of the response. Operationalising the rapid response service is facilitated by the presence of Standard Operating Procedures and templates for preparing and disseminating rapid response products, the availability of a team with appropriate sets of skills and expertise, access to relevant databases, and flexibility in funding to re-orient human, financial and material resources to respond to the pandemic.

### Position the KT platform as a credible source of evidence during a pandemic

At the time of crisis, KT platforms must rapidly position themselves as credible hubs that provide trustworthy evidence to policy-makers, stakeholders, citizens and media. This can be achieved by actively demonstrating the value of the platform early on rather than taking a back seat and waiting for people to turn to them for information. When the crisis hit Lebanon, the K2P Center launched the K2P COVID-19 Series Initiative and the first policy-relevant document produced in that series cemented and reinforced K2P’s role as a trusted reference centre for evidence and guidance related to COVID-19. At the downstream level, the K2P Center activated the ‘K2People’ initiative to educate and raise awareness of citizens and media about the virus and its mode of transmission as well as to address scepticisms and misconceptions through social media platforms. As part of the ‘K2People’ initiative, we sought to target vulnerable groups that are mostly affected by the COVID-19 pandemic such as smokers, cancer patients, elderly population, refugees and low socio-economic workers. We also tackled mental health problems affecting adults as well as children as a result of the COVID-19 pandemic. The abovementioned two initiatives also provided a clear portal for communicating COVID-19 information, which can be accessed for free by all relevant and interested stakeholders.

Importantly, KT platforms must maintain their credibility throughout the crisis by demonstrating a high degree of responsiveness to priorities and needs, remaining objective, truthful and politically neutral, and ensuring transparency in the evidence and recommendations generated. This is particularly relevant for KT platforms established independently or within an academic institution where trust is a pre-requisite to interacting with such platforms.

### Remain alert and responsive to changing priorities and needs, both upstream and downstream

Decision-makers’ needs for evidence can vary, depending on the context, type of stakeholder, resource availability, or the specific phase of the pandemic cycle in a given country [19]. To improve the translation of evidence into policy and practice, it is important for KT platforms to identify context-specific knowledge gaps, priorities and needs and to subsequently address them through a corresponding product (e.g. rapid evidence summary, guidance document, evidence brief, media bite) tailored to the target audience.

Moreover, given the high level of uncertainty associated with such a pandemic, there is a huge appetite for evidence to guide decision-making at all levels. This presents a critical opportunity for KT platforms to not only respond to decision-makers’ priorities and needs, but to also proactively shape the policy agenda by bringing important (often overlooked) issues to the attention of decision-makers. This requires KT platforms to remain vigilant of changes in the health system, closely monitor social media (e.g. through real-time content analysis of tweets) and keep abreast of how the COVID-19 situation is unfolding at the international level. Anticipating the types of needed decisions can help KT platforms prioritise and prepare ahead of time in order to ensure a more timely response to emerging priorities.

Furthermore, given the scale and breadth of COVID-19, a top-down approach will likely fail to achieve the desired impact; organisations, initiatives and networks that support evidence-informedpolicy-making can help balance top-down with bottom-up approaches by catering to the needs not only of governments and policy-makers but also of policy implementers, including municipalities, healthcare providers, civil society organisations and communities. For the latter groups, the lack of clarity on their roles and responsibilities may hinder their involvement in the pandemic response. To overcome this, the K2P Center produced a number of evidence-based guidance documents specifying the roles of the different actors and the link to the national pandemic response. These guidance documents played a role in empowering the different actors and prompted government to strengthen its stewardship function for a more effective and efficient response.

### Search for evidence beyond ‘conventional’ types and sources

During pandemics, evidence-informeddecision-making may be challenged by scientific uncertainties and scarcity of research, especially during the early phases of the crisis. Conventionally, systematic reviews and randomised controlled trials are considered the gold standards for informing decisions on what works. However, in crisis situations, evidence is needed to address a broad range of questions beyond what works. Moreover, the ‘best’ evidence, i.e. the most valid and reliable evidence, may not be available, yet decisions have to be made fast and under great uncertainty. In many instances, there may only be a single case study or an observational study available, rendering these the best available evidence. Furthermore, indirect evidence becomes particularly valuable during the early phases of the pandemic when there is limited research directly addressing COVID-19. Under such circumstances, turning to indirect evidence from closely related viruses like influenza, SARS (severe acute respiratory syndrome) or Ebola virus may be necessary to inform policy response. As such, KT platforms should aim for the best available evidence while acknowledging the tension between rigor and speed; waiting until more research and better data become available may risk decisions being made with no evidence at all due to time constraints.

Unfortunately, evidence at time of crisis is scattered around different databases, journals, websites and in the grey literature [26]. Therefore, the search for the best available evidence should not be restricted to peer-reviewed journals and electronic databases but also to other sources of information such as reports by national and international agencies, governmental websites, social media platforms (e.g. Twitter), media websites, email subscriptions and blogs, and direct correspondence with senior decision-makers and health professionals in the field. This non-traditional way of searching for evidence may be necessary in a context of rapid evolution and complexity with knowledge constantly changing and evolving. Thus, what constitutes evidence in a crisis setting is often broader than the research generated through the ‘scientific method’. The key is to exercise transparency and be explicit about the sources of evidence informing a policy response and acknowledge any limitations and uncertainties in the evidence base.

The emergence of international initiatives like the COVID-19 Evidence Network to support Decision-making (COVID-END) partners, which are compiling COVID-related evidence from partners around the world in one repository, is a much needed step in addressing the fragmentation of the evidence base and reducing the duplication of efforts. Establishing similar initiatives at the country level is much warranted.

### Harness the strength of complementary evidence networks

The highly dynamic trajectory of the COVID-19 pandemic and the large number of intertwined health, social and economic factors associated with it mean that no single entity can provide all the needed support for a comprehensive response to the pandemic. Researchers, public health specialists, guideline developers, epidemiologists, data analysts and evaluation experts from both health and non-health sectors are all generating relevant and timely evidence to inform the various aspects of the pandemic — from controlling the spread of virus to assessing the effectiveness of public health and social interventions to evaluating the impact on health and the economy. Yet, the absence of a mechanism to bridge the different entities can result in ad hoc, fragmented and delayed engagement, which can undermine the efficiency and effectiveness of the pandemic response.

The COVID-19 pandemic has exposed the deficiencies of siloed approaches to informing decision-making processes and re-enforced the need to build bridges to the organisations, institutions and networks working in complementary areas to inform decision-making related to different aspects of the pandemic. Moving forward, it is critical to establish mechanisms for coordinating and integrating research, data and expertise across stakeholders and sectors in transparent ways for a more effective policy response during pandemics.

### Leverage multiple dissemination channels tailored to different audiences

Dissemination of the evidence to target the right audience is crucial to achieve the desirable impact. In a time of pandemic, where conventional dissemination methods like policy dialogues may not be feasible, it is critical for KT platforms to innovate and leverage multiple dissemination channels suited to the context and audience at the right time.

Social media is an increasingly important platform to disseminate evidence from research and knowledge translation products during pandemics [27]. KT platforms should invest in developing a robust social media presence and should establish relationships with key journalists that they can leverage to help disseminate the evidence to a wide range of audiences, including policy-makers, healthcare professionals, non-governmental organisations and the general public. Therefore, KT platforms should have a communication strategy set in place to allow a timely response in times of crisis and emergencies. In addition, media messages, including Twitter and other social media platforms, should be evidence-based yet concise and simple, utilising videos, visuals and infographics whenever possible in order to engage lay people and decision-makers. Television/radio interviews, podcasts and online webinars constitute other ways to disseminate relevant evidence to decision-makers, practitioners and the public during pandemics.

## Conclusions

The response to the COVID-19 pandemic has been challenged by many factors, including scientific uncertainties, scarcity of relevant research, proliferation of misinformation and fake news, poor access to actionable evidence, time constraints, and weak collaboration among relevant stakeholders. This Commentary highlighted the emerging roles of KT platforms in light of the COVID-19 pandemic. The COVID-19 experience is an opportunity to amplify the roles of KT platforms and position them as the go-to hubs for trustworthy evidence that can inform policies and practice during public health crises. The lessons learned reflected in this Commentary can be integrated into strengthening the role, structures and mandates of KT platforms.

In the post COVID-19 era, it is expected that much of the global and national efforts will focus on strengthening public health systems. Organisations, initiatives and networks supporting evidence-informedpolicy-making are an integral and indispensable component of strengthening public health systems and preparedness response; therefore, one of the best investments in the future is to invest in strengthening these entities to support a more proactive and swift response to public health crises that utilises the best available research evidence and data in a timely manner. This calls for countries, governments, science communities and funders to start investing in KT platforms in different regions through securing adequate resources and supporting capacity-building in KT through education and training programmes and, more importantly, promoting their integration and institutionalisation within the policy-making processes. This is particularly relevant in the context of low- and middle-income countries, where the utilisation of evidence in decision-making is still limited and challenged by the lack of importance given to evidence, the poor communication between researchers and decision-makers, corruption, insufficient training, institutional constraints and weak mechanisms to support evidence-informeddecision-making [28, 29].

## Data Availability

Not applicable.

## Abbreviations

COVID-19: coronavirus disease
KT: knowledge translation
K2P Center: Knowledge to Policy (K2P) Center
